# Testing region selection and prognostic analysis of *MLH1* promoter methylation in colorectal cancer in China

**DOI:** 10.1093/gastro/goae011

**Published:** 2024-04-02

**Authors:** Xiaoli Tan, Yongzhen Fang, Xinjuan Fan, Weihao Deng, Jinglin Huang, Yacheng Cai, Jiaxin Zou, Zhiting Chen, Hanjie Lin, Liang Xu, Guannan Wang, Huanmiao Zhan, Shuhui Huang, Xinhui Fu

**Affiliations:** Department of Pathology, The Sixth Affiliated Hospital, Sun Yat-sen University, Guangzhou, Guangdong, P. R. China; Guangdong Provincial Key Laboratory of Colorectal and Pelvic Floor Diseases, The Sixth Affiliated Hospital, Sun Yat-sen University, Guangzhou, Guangdong, P. R. China; Department of Pathology, The Sixth Affiliated Hospital, Sun Yat-sen University, Guangzhou, Guangdong, P. R. China; Department of Pathology, The Sixth Affiliated Hospital, Sun Yat-sen University, Guangzhou, Guangdong, P. R. China; Department of Pathology, The Sixth Affiliated Hospital, Sun Yat-sen University, Guangzhou, Guangdong, P. R. China; Department of Pathology, The Sixth Affiliated Hospital, Sun Yat-sen University, Guangzhou, Guangdong, P. R. China; Guangdong Provincial Key Laboratory of Colorectal and Pelvic Floor Diseases, The Sixth Affiliated Hospital, Sun Yat-sen University, Guangzhou, Guangdong, P. R. China; Department of General Surgery, The Sixth Affiliated Hospital, Sun Yat-sen University, Guangzhou, Guangdong, P. R. China; Department of Pathology, The Sixth Affiliated Hospital, Sun Yat-sen University, Guangzhou, Guangdong, P. R. China; Department of Pathology, The Sixth Affiliated Hospital, Sun Yat-sen University, Guangzhou, Guangdong, P. R. China; Department of Pathology, The Sixth Affiliated Hospital, Sun Yat-sen University, Guangzhou, Guangdong, P. R. China; Guangdong Provincial Key Laboratory of Colorectal and Pelvic Floor Diseases, The Sixth Affiliated Hospital, Sun Yat-sen University, Guangzhou, Guangdong, P. R. China; Department of Pathology, The Sixth Affiliated Hospital, Sun Yat-sen University, Guangzhou, Guangdong, P. R. China; Department of Pathology, The Sixth Affiliated Hospital, Sun Yat-sen University, Guangzhou, Guangdong, P. R. China; Department of Pathology, The Sixth Affiliated Hospital, Sun Yat-sen University, Guangzhou, Guangdong, P. R. China; Biomedical Innovation Center, The Sixth Affiliated Hospital, Sun Yat-sen University, Guangzhou, Guangdong, P. R. China

**Keywords:** CRC, MLH1 deficiency, *MLH1* promoter methylation, overall survival

## Abstract

**Background:**

*MLH1* promoter methylation analysis is recommended in screening for Lynch syndrome (LS) in patients with MLH1-deficient colorectal cancer (CRC). The study aims to identify specific methylation regions in the *MLH1* promoter and to evaluate the clinicopathologic characteristics of and prognosis for patients with *MLH1* methylation.

**Methods:**

A total of 580 CRC cases were included. The DNA mismatch repair (MMR) protein expression was assessed by using immunohistochemistry (IHC). The methylation status of the Regions A, B, C, D, and E in the *MLH1* promoter was tested by using bisulfite sequencing PCR. The specificities of the five regions were calculated. Associations between *MLH1* methylation and clinicopathologic characteristics were evaluated. Kaplan–Meier analyses for overall survival (OS) were carried out.

**Results:**

In 580 CRC cases, the specificities of the methylation test in Regions D and E were both 97.8%. In the MLH1-deficient CRCs, the frequencies of *MLH1* methylation and *BRAF^V600E^* mutation were 52.6% and 14.6%, respectively; *BRAF^V600E^* mutation occurred in 27.7% of patients with *MLH1*-methylated CRC. In the MMR-deficient patients, compared with *MLH1* unmethylation, *MLH1* methylation was more common in patients who were aged ≥50 years, female, had no family history of LS-related tumors, and had tumors located at the right colon. In the MMR-deficient patients, the *MLH1*-methylated cases had lower OS rates than the unmethylated cases with a family history of LS-related tumors (*P *=* *0.047).

**Conclusions:**

Regions D and E in the *MLH1* promoter are recommended for determining the *MLH1* methylation status in screening for LS in MLH1-deficient CRC. In MMR-deficient patients, the *MLH1*-methylated cases had a worse OS than the unmethylated cases with a family history of LS-related cancer.

## Introduction

Colorectal cancer (CRC) is the third most frequently diagnosed cancer in the world [1] and the second in China [2]. A universal recommendation is that all newly diagnosed patients with CRC should be tested for DNA mismatch repair (MMR) or microsatellite instability (MSI) to identify individuals with Lynch syndrome (LS) and to guide clinical therapy [3]. MMR deficiency (dMMR) occurs in ∼10%–15% of all CRCs and 3% of these are associated with LS—an autosomal dominant disorder caused by germline mutations in MMR genes (such as *MLH1*, *MSH2*, *MSH6*, or *PMS2*); the other 12% are sporadic CRC, caused by hypermethylation of the *MLH1* promoter [4, 5]. It is recommended that abnormal MLH1 immunohistochemistry (IHC) in tumor tissues should be followed by testing for *MLH1* promoter methylation or *BRAF^V600^*^E^ mutation [3].

*MLH1* promoter methylation has been widely studied, but the CpG sites in the promoter are detected differently. Deng *et al.* [6] reported in 1999 that methylation of CpG sites in a small region from −269 to −199 before the start codon of the *MLH1* gene (Deng Region C, from −248 to −178 relative to the transcription start site in their study) was most associated with the loss of MLH1 expression by using NaHSO_3_ treatment-sequencing, but it was investigated in CRC cell lines. Miyakura *et al.* [7] reported in 2001 that the methylation of CpG sites in the upstream range (from −755 to −574) is an early event in the carcinogenesis of MSI-H tumors and may arise in normal tissues by using single-strand conformation polymorphism analysis. These pieces of research showed that the upstream range of the *MLH1* promoter is not correlated with the lack of MLH1 expression. Then, Deng *et al.* [8] reported in 2002 that *MLH1* methylation correlates with MLH1 expression in a region-specific manner in CRC when using the NaHSO_3_-digestion method (or the combined bisulfite restriction analysis, COBRA) by using restriction enzyme BstUI that recognizes two consecutive CpG sites (CGCG) from −252 to −249 within this proximal region. However, only nine patients with deficient MLH1 CRC were included. Via new technical advances, a commercial *MLH1* methylation assay kit was widely used [9–13] by using methylation-specific multiplex ligation-dependent probe amplification (MS-MLPA) based on the use of probes that contain one or two digestion sites specific for the methylation-sensitive HhaI enzyme (that recognizes GCGC sites) (http://www.mrc-holland.com). The kit determines *MLH1* methylation status according to the GCGC sites in the Deng Regions C and D (−251 to −248, −245 to −242, and −8 to −5 relative to the start codon). However, the methylation-specific restriction enzyme recognizes limited CpG sites. Although most studies tested the *MLH1* methylation in the Deng Region C, some recent studies still used various CpG sites and methods for *MLH1* methylation detection [14].

In this study, we detected *MLH1* promoter methylation in a large sample of 580 patients with CRC using bisulfite sequencing PCR (BSP). We analysed the specific CpG regions that are highly associated with *MLH1* deficiency in the *MLH1* promoter. We also evaluated the associations between *MLH1* methylation and clinicopathological characteristics, and analysed the prognosis for patients with *MLH1* promoter methylation.

## Materials and methods

### Patients

**Figure 1.**
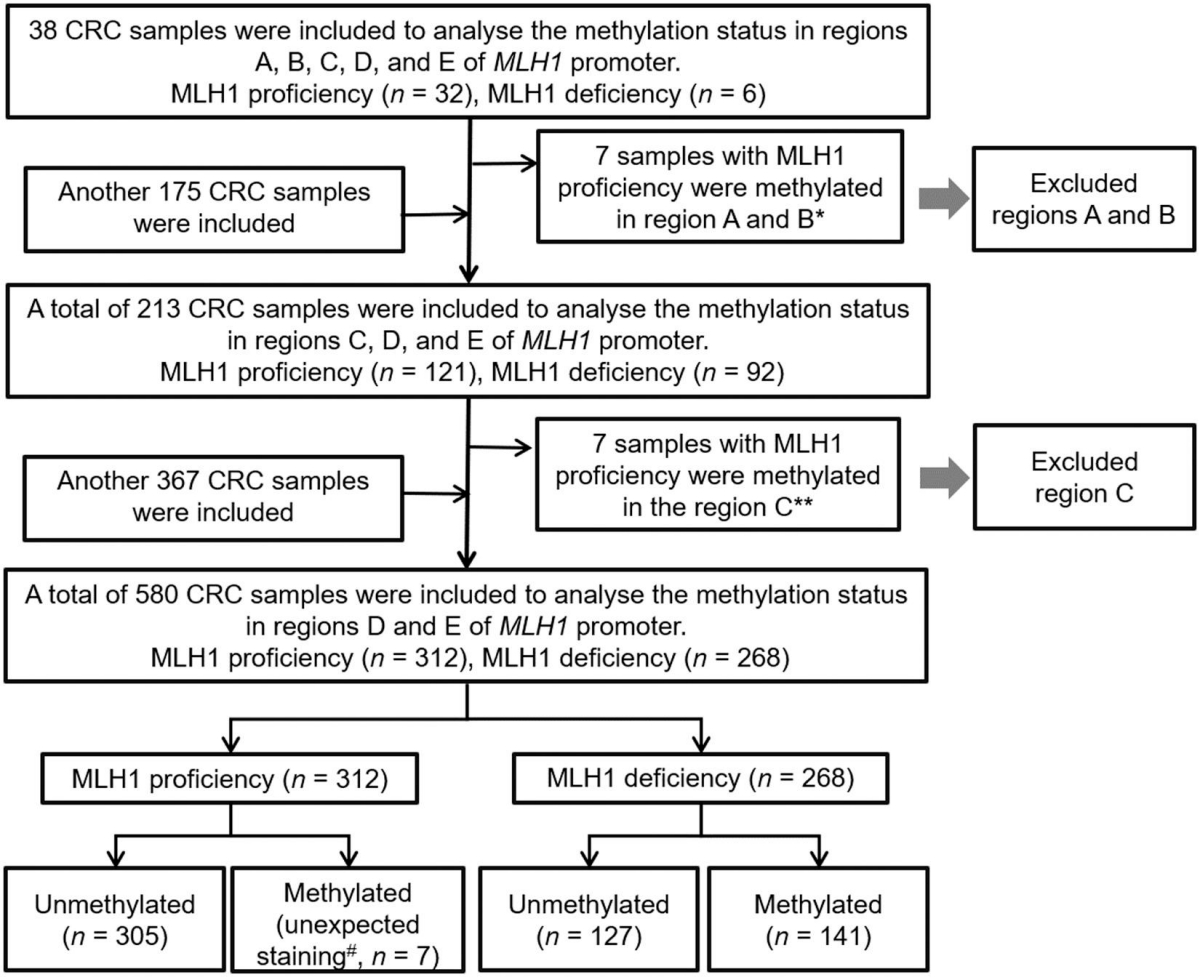
Flow diagram of the analytical strategy. ^*^Seven samples reassessed by two experienced pathologists with MLH1 proficiency were fully or partially methylated in Regions A and B. ^**^Seven samples reassessed by two experienced pathologists with MLH1 proficiency were partially methylated in Region C, including one case that methylated in Regions A and B in the above analysis. ^#^Seven cases with MLH1 proficiency were reassessed by two experienced pathologists presenting unexpected MLH1 staining, such as tumor staining weaker than control or heterogeneous staining. CRC = colorectal cancer.

In the study, the patients were included in a stepwise way and the screening process was as shown in Figure 1. A total of 580 patients with CRC diagnosed at the Sixth Affiliated Hospital, Sun Yat-sen University (Guangzhou, Guangdong, China) from March 2013 to October 2022 were included. Due to the low prevalence of dMMR in Chinese patients with CRC, especially for patients with MLH1/PMS2 deficiency [5, 15], the cohort was selected non-randomly. To obtain abundant representative cases for investigating the methylation status of *MLH1*, dMMR cases were intensively collected according to the IHC results. All the tissue samples in this study were operation specimens. This study was approved by the Ethics Committee of the Sixth Affiliated Hospital, Sun Yat-sen University (approval number: L2017ZSLYEC-003). All patients underwent an informed consent process approved by the Hospital Institutional Review Board.

Colorectal tumor specimens were fixed in formalin, embedded in paraffin after surgery, and confirmed histologically. The clinicopathologic features of these patients were collected from their medical records. A total of 541 patients had available follow-up records, and the follow-up started on the day of surgery and ended on 29 December 2022. The median follow-up time was 29.2 months.

### DNA extraction and bisulfite conversion

Genomic DNA was extracted from formalin-fixed paraffin-embedded (FFPE) samples using a Hipure DNA extraction kit (Cat No. IVD 3126; Magen; Guangzhou, Guangdong, China). Tumor DNA was treated with sodium bisulfite by using the EZ DNA Methylation Kit (Cat No. D5001; ZYMO RESEARCH; Orange County, CA, USA). In brief, 1 μg of DNA was input through the conversion and purification procedure, and then eluted in a 10-μL M-Elution buffer. Meanwhile, CpGenome^TM^ Human Non-Methylated DNA Standard (Cat No. # S8001U; Millipore; Billerica, MA, USA) and CpGenome^TM^ Human Methylated DNA Standard (Cat No. # S8001M; Millipore) were used as controls.

### *MLH1* promoter methylation sequencing

**Figure 2.**
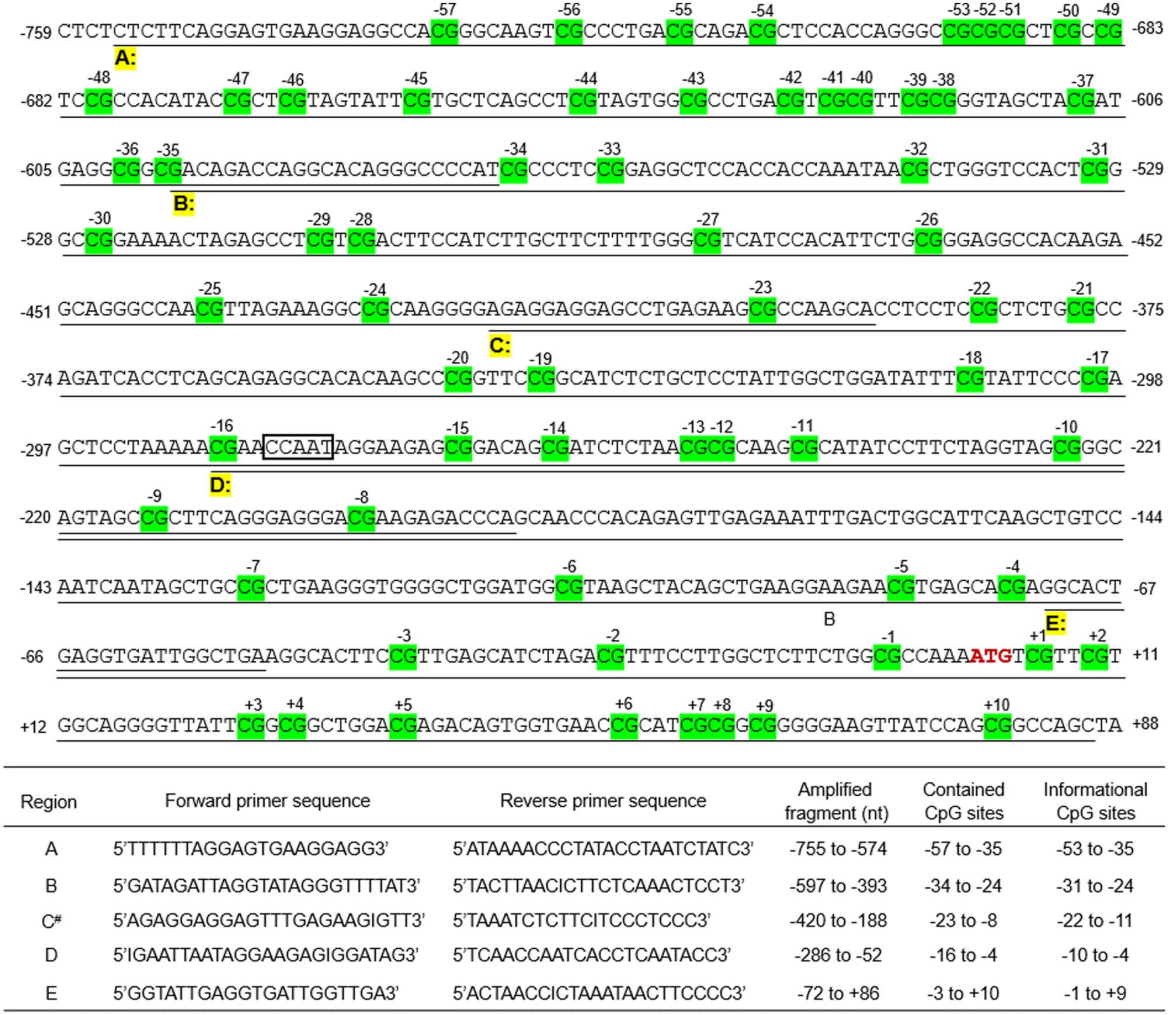
The DNA sequences of the 5’ region of *MLH1*, the primers, and PCR products. The DNA sequences from −759 to +88 in the promoter and exon 1 of *MLH1* (relative to the start codon, NG_007109.2). The CpG sites are marked with a gray background and numbered relative to the start codon. Five primer sets were used to amplify bisulfite-modified DNA and produce five overlapping regions from −755 to +86 [7]. The CCAAT box (–282 to −278 in the promoter) is framed in a box, which specifically binds the transcription factor CBF and affects *MLH1* transcription [16]. # indicates that the reverse primer was used in the sequencing assay of Region C. The forward primer was used in the sequencing assay of Regions A, B, D, and E. CBF = core binding factor.

*MLH1* promoter methylation sequencing was detected using five primer sets that amplified five overlapping regions from −755 to +86 relative to the start codon. The *MLH1* gene promoter was divided into Regions A, B, C, D, and E (Figure 2), according to the article by Miyakura *et al.* [7]. The prior PCR amplification was carried out in 20 μL volume containing 30–50 ng of bisulfite-modified DNA and 8 pmol of each primer (0.4 μM) using an ABI Veriti PCR system, with the following program: initial denaturation at 95°C for 12 min; 40 cycles of denaturation at 95°C for 30 s, annealing at 60°C for 30 s, extension at 72°C for 30 s; final extension at 72°C for 10 min. Then the PCR products were cleaned up and sequenced with a single primer (forward or reverse primer) by using the BigDye Terminator v3.1 Sequencing Standard Kit (Cat No. 4337455; Thermo Fisher Scientific; Waltham, MA, USA) with an ABI Prism 3500Dx Genetic Analyzer (Applied Biosystems; Foster City, CA, USA). All the informational CpG sites in each methylated region were defined as full methylation, partial methylation if there was more than one CpG site in each region methylated, and unmethylation if none of the CpG sites in each region methylated was defined.

### MMR proteins detection

The expression of MMR proteins was detected in all tumor samples by using IHC with a Ventana BenchMark XT autostainer. Before loading slides onto the autostainer, 4-μm tissue sections from FFPE tissue blocks were dried at 65°C for 15 min in a drying oven. Then, the slide samples were immunohistochemically stained with monoclonal antibodies to MMR proteins, including MLH1 (Cat No. MAB-0789; MXB; Fuzhou, Fujian, China), MSH2 (Cat No. IR376, LBP, Guangzhou, Guangdong, China), MSH6 (Cat No. ZA-0541; ZSGB-BIO; Zhongshan, Guangdong, China), and PMS2 (Cat No. ZA-0542; ZSGB-BIO), by using the autostainer. IHC was assessed following the College of American Pathologists (CAP) Colon and Rectum Biomarker Reporting Template [29]. Any positive reaction in tumor cell nuclei would be considered an intact expression. Intact expression of all four MMR proteins indicates MMR proficiency (pMMR) and any loss means dMMR. Adjacent normal tissue and lymphocytes of each sample served as an internal control.

### *BRAF^V600E^* testing

Somatic *BRAF^V600E^* testing was conducted via Sanger sequencing or an allelic discrimination test using an allelic-specific probe. The methods have been described in our previous study [18].

### Statistics analysis

The Spearman chi-square test, Kruskal–Wallis test, and Fisher's exact test were applied to analyse the association of *MLH1* methylation status with clinicopathologic features. The analyses were initially evaluated using continuous variables and categories data analysis, then further accessed using logistic regression models by estimating the odds ratios (ORs) and 95% confidence intervals (CIs). All these statistical analyses were performed using SPSS 22.0 packages (SPSS; Chicago, IL, USA). Kaplan–Meier survival curves for the OS of 541 patients with available follow-up records were performed using GraphPad Prism 8 (Graph Pad Software Inc.; San Diego, CA, USA) via a log-rank test. A *P*-value of <0.05 was considered statistically significant.

## Results

### Patients

This study involved 580 patients with CRC who were selected by using a stepwise method. The clinicopathologic characteristics of this study population were summarized (Table 1). The population's average age was 54.5 years (19–92 years), with a male-to-female ratio of 1.45 to 1, and 7.2% of the cases had a family history of LS-related tumors including CRC, gastric cancer, breast cancer, endometrial cancer, and cholangiocarcinoma. Among all the tumors, 50.5% were located at the right colon, 55.2% were moderately differentiated adenocarcinoma, and 48.5% were diagnosed at the tumor node metastases (TNM) II stage. The cohort contained 172 patients with pMMR and 408 patients with dMMR. Of the patients with dMMR, 259 were MLH1- and PMS2-deficient, 9 were MLH-deficient only, 42 were PMS2-deficient only, and 98 were MSH2- and/or MSH6-deficient. *BRAF^V600E^* mutation was found in 8.3% of the population.

**Table 1.** Demographic and clinical characteristics for the 580 patients with CRC

Characteristic	All patients (*n *=* *580)
Age, years	
Mean (SD)	54.5 (14.8)
Median (range)	56 (17 − 92)
Age, years, *n* (%)	
<50	207 (35.7)
50 − 75	320 (55.2)
>75	53 (9.1)
Sex, *n* (%)	
Female	237 (40.9)
Male	343 (59.1)
Family history, *n* (%)	
Without	538 (92.8)
With	42 (7.2)
Tumor location^a^, *n* (%)	
Rectum	137 (23.6)
Left colon	150 (25.9)
Right colon	293 (50.5)
Differentiation of tubular adenocarcinoma, *n* (%)	
Poor	99 (17.1)
Moderate	320 (55.2)
Well	53 (9.1)
Non-tubular adenocarcinoma	108 (18.6)
TNM stage, *n* (%)	
0	1 (0.2)
I	57 (9.9)
II	280 (48.5)
III	182(31.5)
IV	57 (9.9)
Absent	3
*BRAF^V600E^* status, *n* (%)	
Wild-type	532 (91.7)
Mutant	48 (8.3)
MMR IHC staining, *n* (%)	
pMMR	172 (29.7)
dMMR	408 (70.3)
MLH1 proficiency	140 (24.1)
MSH2 and/or MSH6 deficiency	98
Only PMS2 deficiency	42
MLH1 deficiency	268 (46.2)
MLH1 and PMS2 deficiency	259
Only MLH1 deficiency	9

SD = standard deviation, TNM = tumor node metastases, MMR = mismatch repair protein, IHC = immunohistochemistry, pMMR = mismatch repair proficiency, dMMR = mismatch repair deficiency, CRC = colorectal cancer.

Family history = family history of Lynch syndrome-related tumors including colorectal cancer, gastric cancer, breast cancer, endometrial cancer, and cholangiocarcinoma.

a Rectum: the terminal intestine 12 cm upwards from the anal verge. Left colon: descending colon, sigmoid colon, and rectosigmoid. Right colon: cecum, ascending colon, and transverse colon.

### The methylation status in Regions A, B, C, D, and E of the *MLH1* promoter

We tested five overlapping regions (A, B, C, D, and E) in the *MLH1* promoter. The landscape of the CpG sites in the *MLH1* promoter is shown in Figure 2. The CpG sites were labeled relative to the start codon. The analytical process is shown in Figure 1.

Firstly, 38 samples, including 32 samples with MLH1 proficiency and 6 samples with MLH1 deficiency, were tested for the methylation status in five regions of the *MLH1* promoter. The results showed that seven samples with MLH1 proficiency were fully methylated in Region A and fully or partially methylated in Region B, of which one sample was partially methylated in Region C (Table 2, Supplementary Table S1, and Supplementary Figure S1). Two experienced pathologists reassessed the samples and confirmed them as intact MLH1 expression (data not shown). The specificities of Regions A, B, C, D, and E in *MLH1* methylation were 78.1%, 78.1%, 96.9%, 100%, and 100%, respectively. Hence, the false positive rate of Regions A and B would be 21.9%. It indicated that the methylation in Regions A and B of the *MLH1* promoter did not directly result in MLH1 deficiency. Then, we analysed the methylation status of Regions C, D, and E in 213 samples, including the above 38 samples. The results showed seven samples with MLH1 proficiency were methylated at the first two CpG sites in Region C, including one that was methylated in Regions A and B (Table 2 and Supplementary Table 1). In the same way, these samples were reassessed and confirmed as intact MLH1 expression (data not shown). The specificity of Regions C, D, and E in *MLH1* methylation were 94.2%, 100%, and 100%, respectively (Table 2). The methylation status in Regions D and E was consistently associated with MLH1 expression.

**Table 2.** Comparisons of the methylation status in Region A, B, C, D, and E of the *MLH1* promoter and MLH1 expression

Region	Methylation status	Analysis of Regions A/B/C/D/E (*n *=* *38)		Analysis of Regions C/D/E (*n *=* *213)^a^		Analysis of Regions D/E (*n *=* *580)^a^	
		MLH1 deficiency	MLH1 proficiency	MLH1 deficiency	MLH1 proficiency	MLH1 deficiency	MLH1 proficiency
		*n* (%)	*n* (%)	*n* (%)	*n* (%)	*n* (%)	*n* (%)
			(95% CI)		(95% CI)		(95% CI)
A	Positive	5 (83.3)	7 (21.9)	–	–	–	–
	Negative	1 (16.7)	25 (78.1) (60.96 − 89.27)	–	–	–	–
B	Positive	5 (83.3)	7 (21.9)	–	–	–	–
	Negative	1 (16.7)	25 (78.1) (60.96 − 89.27)	–	–	–	–
C	Positive	5 (83.3)	1 (3.1)	49 (53.3)	7 (5.8)	–	–
	Negative	1 (16.7)	31 (96.9) (82.89 − 99.99)	43 (46.7)	114 (94.2) (88.34–97.37)	–	–
D	Positive	5 (83.3)	0	49 (53.3)	0	141 (52.6)	7 (2.2)^b^
	Negative	1 (16.7)	32 (100) (87.27 − 100.00)	43 (46.7)	121 (100) (96.30–100.00)	127 (47.4)	305 (97.8) (95.35–99.00)
E	Positive	5 (83.3)	0	49 (53.3)	0	141 (52.6)	7 (2.2)^b^
	Negative	1 (16.7)	32 (100) (87.27 − 100.00)	43 (46.7)	121 (100) (96.30–100.00)	127 (47.4)	305 (97.8) (95.35–99.00)

The specificities of Regions A, B, C, D, and E in 38 samples were 78.1%, 78.1%, 96.9%, 100%, and 100%.

The specificities of Regions C, D, and E in 213 samples were 94.2%, 100%, and 100%.

The specificities of Regions D and E in 580 samples were 97.8%.

CI = confidence interval.

a The cohort contained the cases included in the previous section.

b Seven cases with MLH1 proficiency were re-reviewed and showed unexpected MLH1 staining.

**Figure 3.**
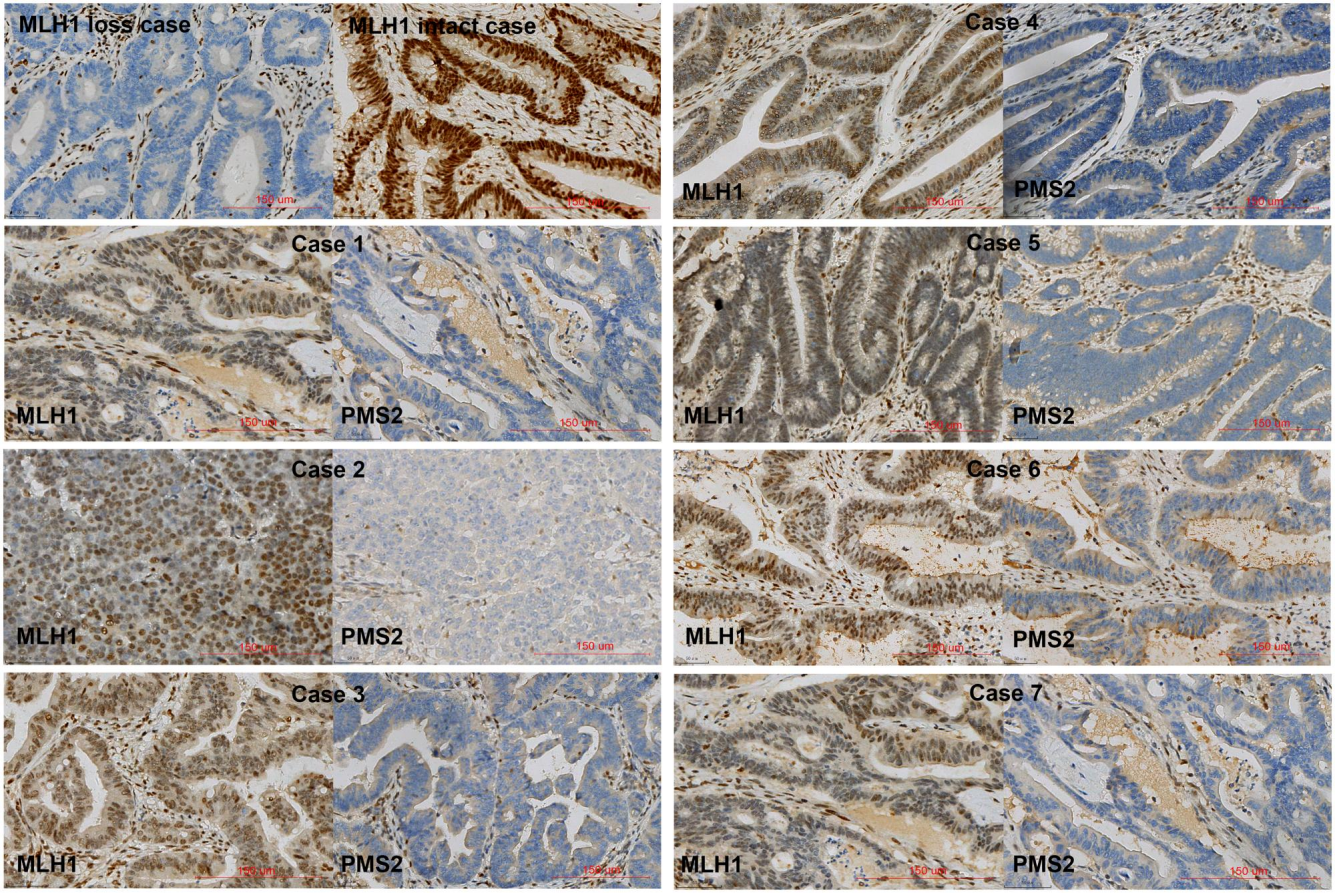
Immunohistochemistry staining of MLH1 and PMS2. MLH1 staining in an MLH1 loss case and an MLH1 intact case (upper left), and MLH1 and PMS2 staining in seven cases with unexpected MLH1 expression, presenting tumor staining weaker than control or heterogeneous staining

Finally, the sample size was expanded to a total of 580 cases and the results confirmed that the methylation status of Regions D and E in the *MLH1* promoter was consistent. Here, seven specimens diagnosed as MLH1 proficiency showed *MLH1* methylation, which displayed PMS2 deficiency. Re-review of MLH1 IHC showed unexpected IHC staining patterns, presenting as heterogeneous status or weak staining in tumor cells (Figure 3). These unexpected MLH1 IHC staining samples were diagnosed as MLH1 proficiency, which led to a specificity of 97.8% in both Regions D and E of the *MLH1* methylation. The methylation status of the *MLH1* promoter was determined according to the result in Regions D and E of the *MLH1* promoter in the study.

### The associations between clinicopathologic characteristics and *MLH1* methylation status

The associations between clinicopathologic characteristics and *MLH1* methylation status were analysed in 408 dMMR patients and 268 MLH1-deficient patients (Tables 3 and 4).

**Table 3.** The associations between *MLH1* methylation status and clinicopathological characteristics in patients with dMMR CRC or MLH1-deficiency CRC

Characteristic	dMMR (*n *=* *408)		*P*	MLH1 deficiency (*n *=* *268)		*P*
	Unmethylated	Methylated		Unmethylated	Methylated	
	(*n *=* *260)	(*n *=* *148)		(*n *=* *127)	(*n *=* *141)	
Age, years			<0.001^b^			<0.001^b^
Mean (SD)	49.6 (13.0)	61.1 (16.7)		49.7 (12.1)	60.9 (16.4)	
Median (range)	50 (19 − 82)	63 (17 − 92)		50 (23 − 82)	63 (17 − 92)	
Age, years, *n* (%)			<0.001			<0.001^c^
<50	125 (48.1)	34 (23.0)		61 (48.0)	33 (23.4)	
50 − 75	128 (49.2)	81 (54.7)		63 (49.6)	78 (55.3)	
>75	7 (2.7)	33 (22.3)		3 (2.4)	30 (21.3)	
Sex, *n* (%)			<0.001			<0.001
Female	89 (34.2)	82 (55.4)		42 (33.1)	77 (54.6)	
Male	171 (65.8)	66 (44.6)		85 (66.9)	64 (45.4)	
Family history, *n* (%)						<0.001^c^
Without	221 (85.0)	147 (99.3)	<0.001^c^	110 (86.6)	140 (99.3)	
With	39 (15.0)	1 (0.7)		17 (13.4)	1 (0.7)	
Tumor location^a^, *n* (%)			0.003			0.036
Rectum	38 (14.6)	13 (8.8)		15 (11.8)	12 (8.5)	
Left colon	74 (28.5)	26 (17.6)		38 (29.9)	26 (18.4)	
Right colon	148 (56.9)	109 (73.6)		74 (58.3)	103 (73.0)	
Differentiation of tubular adenocarcinoma, *n* (%)			0.223			0.099
Poor	45 (17.3)	38 (25.7)		21 (16.5)	37 (26.2)	
Moderate	135 (51.9)	70 (47.3)		74 (58.3)	66 (46.8)	
Well	26 (10.0)	11 (7.4)		13 (10.2)	10 (7.1)	
Non-tubular adenocarcinoma	54 (20.8)	29 (19.6)		19 (15.0)	28 (19.9)	
TNM stage, *n* (%)			0.932			0.531
I	25 (9.7)	14 (9.5)		12 (9.5)	14 (9.9)	
II	151 (58.3)	82 (55.4)		80 (63.5)	79 (56.0)	
III	68 (26.3)	42 (28.4)		27 (21.4)	41 (29.1)	
IV	15 (5.8)	10 (6.8)		7 (5.6)	7 (5.0)	
Absent	1			1		
*BRAF^V600E^* status, *n* (%)			/			/
Wild-type	260 (100)	106 (71.6)		127 (100)	102 (72.3)	
Mutant	0	42 (28.4)		0	39 (27.7)	

SD = standard deviation, TNM = tumor node metastases, dMMR = mismatch repair deficiency, CRC = colorectal cancer.

Family history = family history of Lynch syndrome-related tumors including colorectal cancer, gastric cancer, breast cancer, endometrial cancer, and cholangiocarcinoma.

a Rectum: the terminal intestine 12 cm upwards from the anal verge. Left colon: descending colon, sigmoid colon, and rectosigmoid. Right colon: cecum, ascending colon, and transverse colon.

Spearman chi-square test.

b Kruskal–Wallis test.

c Fisher's exact test.

**Table 4.** Logistic regression model associations between clinicopathologic characteristics and *MLH1* methylation in patients with dMMR or MLH1 deficiency

Characteristic	MLH1 methylation in dMMR patients (*n *=* *408)				MLH1 methylation in MLH1 deficient patients (*n *=* *268)			
	Univariate analysis		Multivariate analysis		Univariate analysis		Multivariate analysis	
	OR (95% CI)	*P*	OR (95% CI)	*P*	OR (95% CI)	*P*	OR (95% CI)	*P*
Age ≥50 years	3.11 (1.97 − 4.89)	<0.001	2.59 (1.60 − 4.18)	<0.001	3.03 (1.79 − 5.10)	<0.001	2.83 (1.64 − 4.90)	<0.001
(vs <50 years)								
Female	2.39 (1.58 − 3.61)	<0.001	2.14 (1.38 − 3.33)	0.001	2.44 (1.48 − 4.00)	<0.001	2.22 (1.31 − 3.76)	0.003
(vs male)								
Without family history	25.94 (3.53 − 190.89)	0.001	19.28 (2.59 − 143.70)	0.004	21.64 (2.84 − 165.10)	0.003	17.39 (2.22 − 136.23)	0.007
(vs others)								
Right colon	2.12 (1.36 − 3.29)	0.001	1.68 (1.05 − 2.70)	0.031	1.94 (1.16 − 3.24)	0.011		
(vs others)								

dMMR = mismatch repair deficiency, OR = odds ratio, CI = confidence interval.

Family history = family history of Lynch syndrome-related tumors including colorectal cancer, gastric cancer, breast cancer, endometrial cancer, and cholangiocarcinoma.

In the cohort of 408 dMMR patients, compared with *MLH1* unmethylation, *MLH1* methylation was more likely to be seen in patients ≥50 years old (77.0% vs 51.9% for patients <50 years old; multivariate OR, 2.59; 95% CI, 1.60–4.18; *P *<* *0.001), females (55.4% vs 34.2% males; multivariate OR, 2.14; 95% CI, 1.38–3.33; *P *=* *0.001), patients without a family history (99.3% vs 85.0% for patients with a family history; multivariate OR, 19.28; 95% CI, 2.59–143.70; *P *=* *0.004), and tumors located at the right colon (73.6% vs 56.9% for tumors located at the rectum and the left colon; multivariate OR, 1.68; 95% CI, 1.05–2.70; *P *=* *0.031). In patients with dMMR, the overall *BRAF^V600E^* mutations occurred in tumors with *MLH1* methylation.

In the patients with MLH1 deficiency, the frequency of *MLH1* methylation was 52.6% (141/268). *MLH1* methylation occurred more frequently in patients aged ≥50 years (76.6% vs 52.0% for patients aged <50 years; multivariate OR, 2.83; 95% CI, 1.64–4.90; *P *<* *0.001), females (54.6% vs 33.1% males; multivariate OR, 2.22; 95% CI, 1.31–3.76; *P *=* *0.003), and patients without family history (99.3% vs 86.6% for patients with a family history; multivariate OR, 17.39; 95% CI, 2.22–136.23; *P *<* *0.001) compared with *MLH1* unmethylation. In the group of MLH1-deficient CRC, the patients with *BRAF^V600E^* mutation were all *MLH1*-methylated (27.7% vs 0 for *MLH1* unmethylated).

### Prognostic effect of *MLH1* methylation in CRC patients

**Figure 4.**
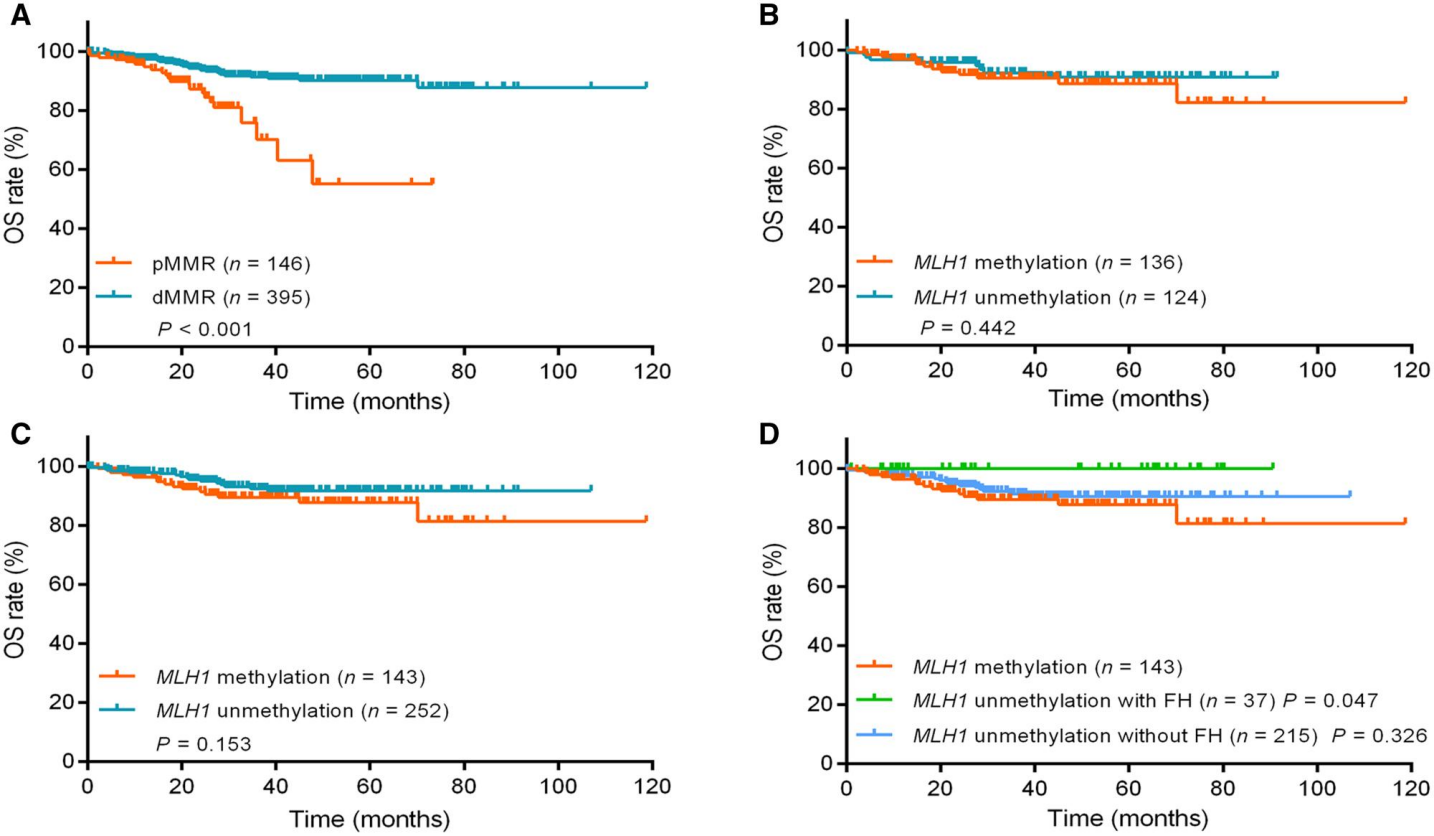
Kaplan–Meier curves of OS for CRC patients with and without *MLH1* methylation. (A) Patients with pMMR vs dMMR. (B) In patients with MLH1 deficiency, patients with *MLH1* methylation vs unmethylation, the unmethylation subgroup included 17 cases with a family history of LS-related cancer. (C) In patients with dMMR, patients with *MLH1* methylation vs unmethylation, the unmethylation subgroup included 37 cases with a family history of LS-related cancer. (D) In patients with dMMR, patients with *MLH1* methylation vs unmethylation with family history. OS = overall survival, CRC = colorectal cancer, pMMR = mismatch repair proficiency, dMMR = mismatch repair deficiency, FH = family history of Lynch syndrome-related tumors including colorectal cancer, gastric cancer, breast cancer, endometrial cancer, and cholangiocarcinoma.

In the study, 541 patients got complete follow-up information. During the follow-up, 53 patients died, of whom 42 died from CRC or related diseases. Kaplan–Meier survival analysis for OS was performed based on MMR and *MLH1* methylation status within different subgroups (Figure 4). The results showed that patients with dMMR had higher OS rates than those with pMMR (*P < *0.001, Figure 4A). In patients with dMMR, *MLH1-*methylated patients had lower OS rates than unmethylated cases with a family history of LS-related cancer (*P = *0.047, Figure 4D). However, there was little difference between the patients with *MLH1* methylation and *MLH1* unmethylation, in patients with either MLH1 deficiency or dMMR, with no significance (*P *=* *0.442, Figure 4B; *P *=* *0.153, Figure 4C). Note that there were 17 and 37 patients with a family history of LS-related cancer in the *MLH1*-unmethylated subgroup in MLH1-deficient patients (Figure 4B) and dMMR patients (Figure 4C), respectively. No patient had family history in the *MLH1*-methylated subgroup.

## Discussion

Based on a large clinical cohort and a wide detection range of the *MLH1* promoter, we identified that the CpG sites in Regions D and E of the *MLH1* promoter have the highest specificity in the *MLH1* methylation test. Regions D and E can be used for *MLH1* methylation detection. This study provides a reference for *MLH1* promoter methylation detection sites in LS screening. Consistently with the previous research [6, 7], we showed that methylation in the upstream range (Regions A and B) of the *MLH1* promoter was not correlated with MLH1 deficiency. In the study, Regions D and E in the *MLH1* promoter contained the Deng Region C and Deng Region D and spread to exon 1 of the *MLH1.* Deng *et al.* identified a CCAAT box in Region D (Figure 2), which specifically bound transcription factor core binding factor (CBF) [16]; they found that methylation at adjacent CpG sites of the CCAAT box inhibited the binding of CBF to the CCAAT box and resulted in the inhibition of the transcription of *MLH1*. It was also reported that *MLH1* promoter methylation spread from intron 1 of the *MLH1* gene to the 5’ region of the *MLH1*, including Regions D and E, resulting in decreased MLH1 expression [17].

In this study, *MLH1* methylation was found in 52.6% (141/268) of MLH1-deficient cases. This percentage is much higher than that in a previous Chinese study (36.7%) [30], but similar to that another study in China (52.7%) [20]. At the same time, *BRAF^V600E^* mutation was found in 14.6% (39/268) of MLH1-deficient cases, which was consistent with previous Chinese studies, at 15.4% or 17.2% [19, 20], but higher than in two other Chinese studies (5.5% and 9.9%, respectively) [5, 30]. In this study, *BRAF^V600E^* mutation occurred in 27.7% (39/141) of patients with *MLH1*-methylated CRC. A previous study reviewed 35 studies and found that *BRAF^V600E^* mutation occurred in 63.5% (95% CI, 46.98%−78.53%) of CRC patients who exhibited *MLH1* methylation or MLH1 loss in Western countries [14]. Guidelines recommend that patients with MLH1 deficiency should be tested for *BRAF^V600E^* or *MLH1* methylation to rule out LS [3]. The above data showed that the *BRAF^V600E^* mutation is a poor surrogate for detecting *MLH1* methylation in China because of its low incidence in *MLH1*-methylated patients. However, it is often used instead for *MLH1* methylation testing because it is cheap. Therefore, we suggest that, except for the *BRAF^V600E^* test, Chinese patients with MLH1-deficient CRC should have more attention paid to *MLH1* methylation testing to rule out LS. Moreover, according to the guidelines, 141 of the 268 cases (52.6%) with MLH1 deficiency would be excluded from germline testing. However, the remainder (47.4%, 127/268) must undergo germline testing to screen for LS. Remarkably, caution should be exercised in excluding cases with strong evidence of germline mutation, despite *BRAF^V600E^* mutation or *MLH1* methylation, based on the observations of *BRAF^V600E^* mutation or *MLH1* promoter methylation in germline *MLH1* mutation carriers [20, 21, 22]. *MLH1* methylation as the “second hit” in the carriers is possible [23]. Further, increasing evidence for the role of constitutional *MLH1* methylation in LS has been found [24–27]. It is suggested that CRC patients aged ≤55 years with tumor *MLH1* methylation should be tested for constitutional *MLH1* methylation before being excluded as a non-LS, although it is rare overall [28].

Another important finding of this study is that the methylation of Regions D and E in the *MLH1* promoter was found in unexpected MLH1 staining tumors, such as tumor staining weaker than control or heterogeneous staining in the tumor nuclei. In the present study, the IHC staining was assessed following the CAP Biomarker Reporting Template [29], which points out that any positive reaction in tumor cell nuclei would be considered as intact expression when a positive reaction is seen in internal control cells. However, the cut-off value for what is considered intact MMR staining has yet to be agreed upon [31, 32]. A key measure of MMR staining is that staining in the tumor nuclei must be equal to or stronger than the internal control [33]. In Figure 3, MLH1 IHC staining of these cases was reassessed and showed unexpected IHC staining patterns, presenting tumor staining that was weaker than the control or heterogeneous staining. Such unexpected IHC staining patterns have been noticed in several laboratories, and most of the aberrant staining patterns are thought to be related to technical issues [33–35]. We also assumed that heterogeneous *MLH1* methylation status in different tumor cells may contribute to MLH1 heterogeneous staining. Obviously, the unexpected staining pattern being immediately interpreted as an intact expression is not appropriate, so it was suggested to either repeat the stain or choose an additional test such as MSI-PCR [32]. Here, we recommend that CRC patients with MLH1 heterogeneous, weak staining, or other unexpected staining patterns in tumor cells should either have the stain repeated and/or an *MLH1* promoter methylation test carried out.

In addition, we found that, in dMMR patients, patients with *MLH1* methylation had a worse OS than unmethylated cases with a CRC-related family history, similarly to the previously published study about endometrial cancer [36]. The observations indicated that *MLH1*-methylated sporadic cases had significantly worse OS than “suspected-LS” cases. The reason why the results show slightly different OS between *MLH1*-methylated sporadic cases and unmethylated dMMR or MLH1-deficient cases may be due to the considerable amount of censoring data during follow-up.

The limitation of this study is that a few CpG sites could not be identified in our sequencing results because of unidirectional sequences with a forward or a reverse primer in the BSP sequencing, and a few CpG sites are located in primers that were not detected.

## Conclusions

In summary, we found that the methylation status in Regions D and E of the *MLH1* promoter was consistent and had higher specificity than other regions in the *MLH1* methylation test based on a large sample size. Regions D and E can be used for *MLH1* methylation detection. *MLH1* methylation and *BRAF^V600E^* mutation were found in 52.6% and 14.6% of the MLH1-deficient cases, respectively, and all MLH1-deficient cases with *BRAF^V600E^* mutation were *MLH1*-methylated. In dMMR patients, *MLH1*-methylated patients had a worse OS than unmethylated cases with a CRC-related family history.

## Authors' Contributions

X.T. and Y.F. selected samples; acquired, analysed, and interpreted the data; and drafted the manuscript. X.F., W.D., J.H., Y.C., J.Z., Z.C., H.L., L.X., G.W., H.Z., and S.H. collected patients’ information, acquired and analysed the data, and revised the manuscript. X.F. conceived, designed, and supervised the study, and revised and finalized the manuscript. All authors read and approved the final manuscript.

## Supplementary Material

goae011_Supplementary_Data
