# Supplementary figures and images for: Testing region selection and prognostic analysis of MLH1 promoter methylation in colorectal cancer in China
Source: Gastroenterol Rep (Oxf). 2024 Apr 2;12:goae011. doi: 10.1093/gastro/goae011 (PMC10985700; doi:10.1093/gastro/goae011)

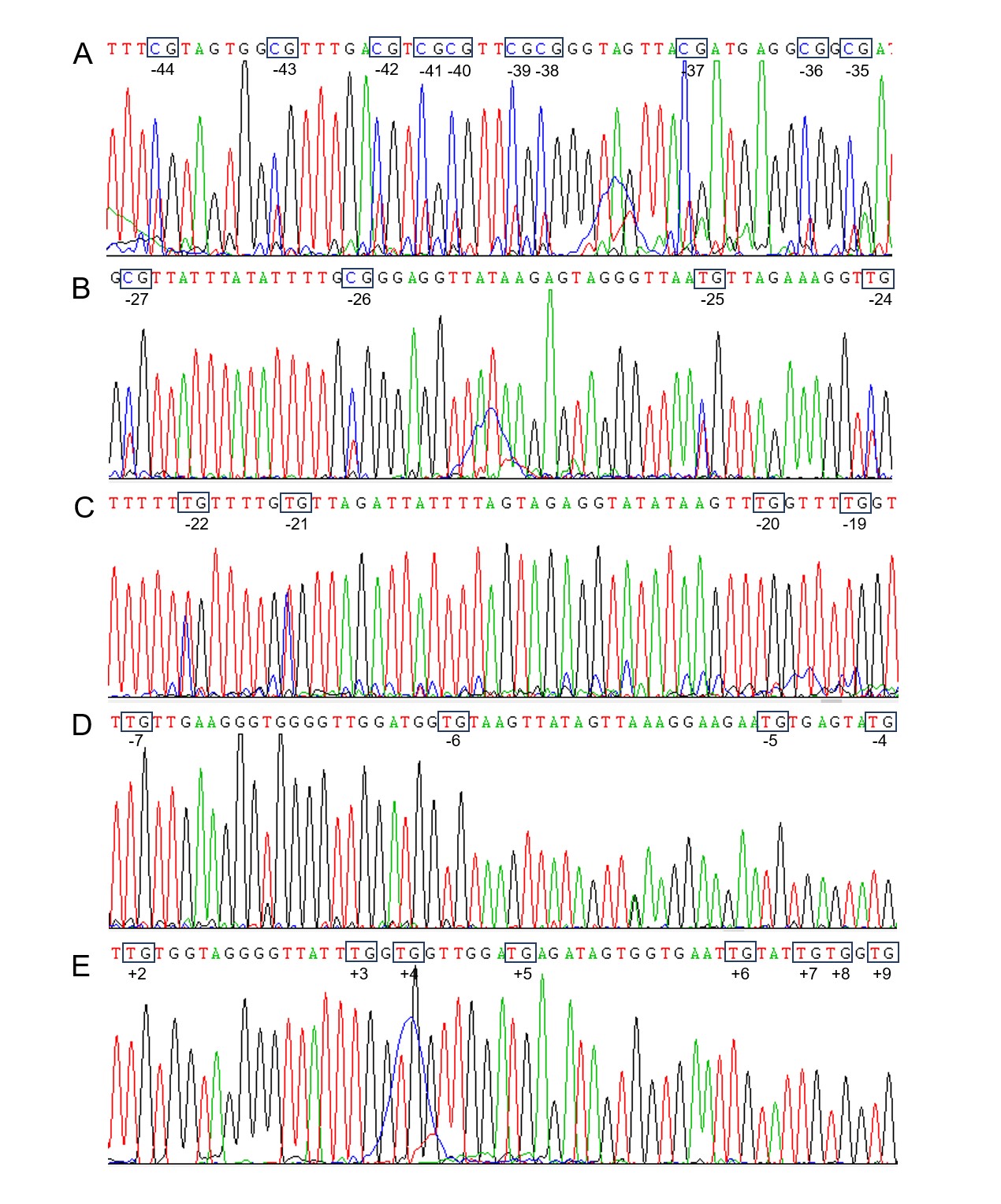

Supplement: goae011_Supplementary_Data [file goae011_supplementary_data.jpeg]
